# Assessing the Efficacy of Anastomosis between Ansa Cervicalis and Facial Nerve for Patients with Concomitant Facial Palsy and Peripheral Neuropathy

**DOI:** 10.3390/jpm13010076

**Published:** 2022-12-29

**Authors:** Georgeta Magdalena Balaci, Aurel Oṣlobanu, Zoltán Zsigmond Major, Réka Emma Dávid, Maria Iepure, Daniela Hancu, Adina Mihaela Popa, Ioan Ṣtefan Florian

**Affiliations:** 1 Department of Neuroscience, Iuliu Hatieganu University of Medicine and Pharmacy, 400347 Cluj-Napoca, Romania; 2 Department of Neurosurgery, Emergency Clinical County Hospital, 400012 Cluj-Napoca, Romania; 3 Department of Neurology, Municipal Clinical Hospital, Tabacarilor Street 11, 400139 Cluj-Napoca, Romania; 4 Department of Neurophysiology, National Center for Spinal Disorders, Királyhágó Street 1, 1126 Budapest, Hungary; 5 Department of Radiology, Emergency Clinical County Hospital, 400006 Cluj-Napoca, Romania; 6 Department of Neurosurgery, Divison of Neurosurgical Anesthesiology, Emergency Clinical County Hospital, 400012 Cluj-Napoca, Romania

**Keywords:** facial nerve, facial palsy, ansa cervicalis, anastomosis, peripheral neuropathy

## Abstract

**Background:** For decades, patients with facial asymmetry have experienced social interaction difficulties, leading them to seek treatment in the hope of restoring facial symmetry and quality of life. Researchers evaluated numerous surgical techniques, but achieving results remains a significant hurdle. Specifically, anastomosis between the ansa cervicalis (AC) and facial nerve (FN) can hinder the patient’s physical appearance. **Objective:** Our study goal was to examine the efficiency of anastomosis between AC and FN for facial motor function recovery even in the presence of peripheral neuropathy. **Materials and Methods**: Four patients diagnosed with facial palsy grade VI on the House & Brackmann Scale (HB) after vestibular schwannoma (VS) resection (Koos grade IV) via the retrosigmoid approach underwent AC and FN anastomosis. Outcomes were related to tumor grade, previous therapy, and the time between postoperative facial palsy and anastomosis. Images and neurophysiological data were evaluated. **Results**: After vs. resection, all four patients demonstrated HB grade VI facial palsy for an average of 17 months. During the follow-up program, lasting between 6 and 36 months, two patients were evaluated as having HB grade III facial palsy; the other two patients were diagnosed with grade IV HB facial palsy. None of the patients developed tongue atrophy, speech disorder, or masticatordys function. **Conclusions**: Anastomosis between the AC and FN is a safe and effective treatment for facial paralysis after cerebellopontine tumor resection. Nerve reanimation may be feasible even for patients with peripheral polyneuropathy. This study also offers a new option for patients with a progression-free status.

## 1. Introduction

High-grade peripheral facial palsy (IV-VI HB) and the risk of keratitis following vestibular schwannoma (VS) surgery can lead to physical and mental suffering for the patient. The goal of facial palsy repair has always been the restoration of facial nerve (FN) function [1,2,3]. For decades, a variety of surgical techniques have been performed to reestablish the paralyzed mimetic muscles using the transfer of muscles such as the gracilis muscle [4] and latissimus dorsi [5] or of nerves such as the nerves that innervate the masseter muscle [6,7] and the hypoglossal [8,9,10], contralateral facial [11,12], and accessory nerves [13,14]. Due to its motor source, the hypoglossal nerve is most frequently used to restore facial motor functions [15,16]. Although this nerve has excellent motor potential, complications such as ipsilateral tongue palsy and swallowing dysfunctions [17] that occur after anastomosis should not be overlooked. Considering the problematic complications of the anastomosis, Ayato et al. [18] applied a new surgical technique, described in 1995 [19], which entails 50% longitudinal sectioning of the hypoglossal nerve diameter for direct epineural neurorrhaphy with the FN. The VII cranial nerve improvement varied from grade V to grade II, and tongue atrophy was minimal to moderate.

Although previous studies [20] have demonstrated excellent results for mid- and lower-face reanimation, Kukva et al. [21] proposed a new technique wherein the hypoglossal nerve is utilized for anastomosis with FN and ansa cervicalis (AC) with simultaneous myoplasty (temporal muscle transposition) for immediate advantages against corneal opacities, low degrees of ipsilateral tongue paralysis, and good results in facial reanimation. The ansa cervicalis, the descending (superior) hypoglossal root, is a reasonable option and is frequently used for facial reanimation to prevent the consequences of tongue hemiatrophy [22], thus avoidingcomplications related to swallowing disorders.

Factors contributing to the development of vs. after a primary malignant tumor (breast, colon, prostate, stomach, and lung) are relatively unknown [23]. The mechanism underlying chemotherapy-induced peripheral neuropathy (CIPN), a complication of oncological treatment involved in the development of hypoesthesia characterized by deficient axonal transport in motor fiber, is yet undefined. Although the incidence of CIPN is estimated to be between 30 and 55%, over 90% of breast cancer patients and 15–30% of patients diagnosed with melanoma or uterine carcinoma develop a symptomatic form of neurotoxicity [24,25,26,27]. The prevalence of peripheral neuropathy in endocrine disorders has been found to be 30–60%. Nerve degeneration, axonal breakdown, and disintegration of neurofilaments represent the underlying mechanism in chronic hypothyroidism, leading to decreased nerve conduction of the compound muscle and sensory nerve action potentials [28,29].

The primary goal of the current study was to analyze the efficiency of anastomosis between the AC and FN in the presence of peripheral neuropathy. As a secondary goal, the role of this anastomosis on facial paralysis recovery without subsidiary effects was reviewed. To the best of our knowledge, these topics have yet to be investigated.

## 2. Materials and Methods

### 2.1. Patient Selection

**Figure 1:**
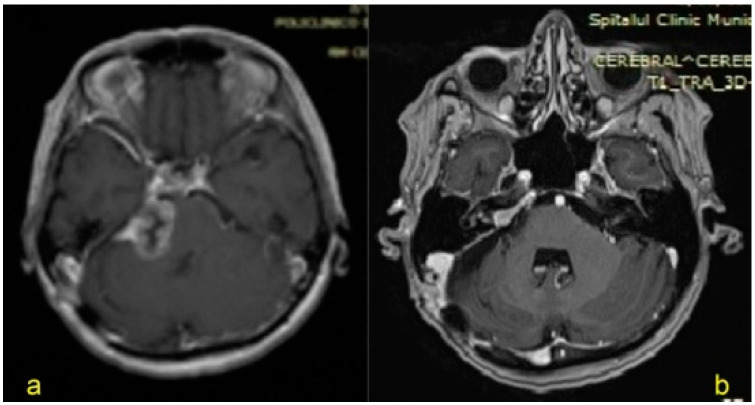
Right vestibular schwannoma, 1st patient, preoperatory (**a**), and prior to nastomosis (**b**).

**Figure 2:**
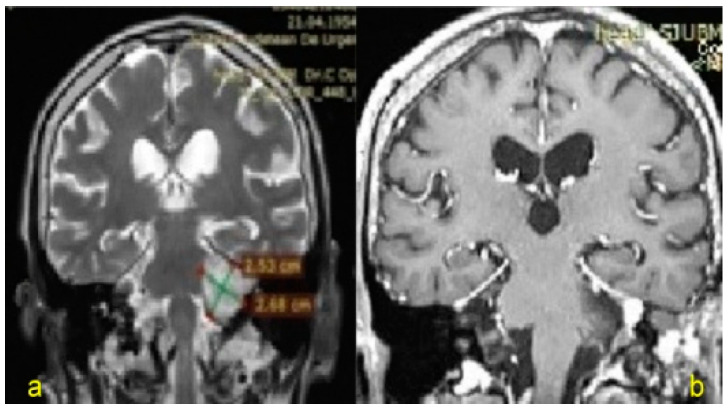
Left vestibular schwannoma, 3rd patient, preoperatory (**a**), and prior to anastomosis (**b**).

According to ethical standards, the present research was authorized along with the assistance of our Institutional Education Committee following the 1964 Helsinki statement and its later modifications. This is a descriptive case series of the first 4 patients (3 women and 1 man) who underwent surgery for complete peripheral facial palsy utilizing anastomosis between the AC and FN. Patients provided written approval for both the surgical technique and the follow-up protocol and were notified of both the benefits and the limits of this technique, taking into consideration peripheral neuropathy. Patients were included in the present report if they met the following criteria:

(1) Surgery for nonneoplastic cerebellopontine tumors and development of high-grade peripheral facial palsy.
(2) No improvement in high-grade facial palsy (HB grades IV-VI) for 6 months following vs. resection based on clinical examination at rest and during voluntary contractions and on neurophysiological monitoring.
(3) Contrast-enhanced brain magnetic resonance imaging (MRIdemonstrating the precise resection of the vs. and excluding any lesion that could affect the hypoglossal nerve or the brainstem (Figure 1 and Figure 2)).
(4) Progression-free status for previousmalignancies.

**Figure 3:**
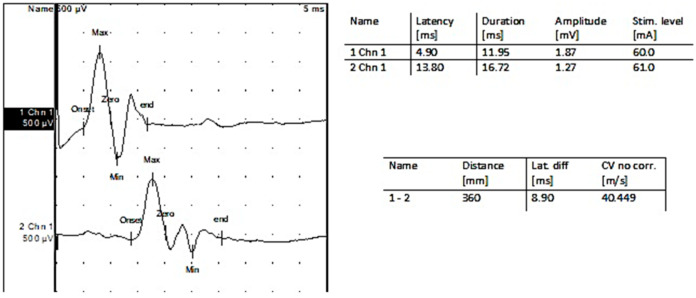
Motor nerveconduction: Left deepperonealnerve.

**Figure 4:**
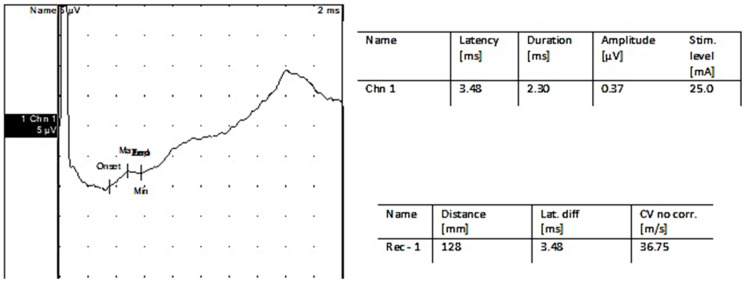
Sensory nerve conduction: Left superficial peroneal nerve.

**Figure 5:**
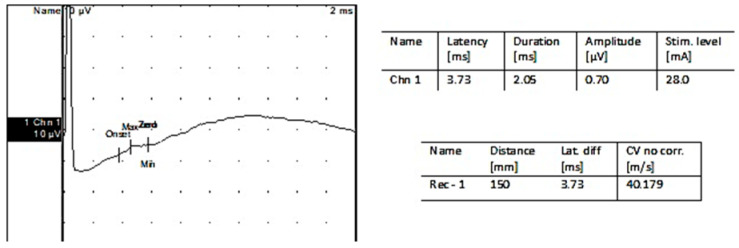
Sensorynerveconduction: Left suralnerve.

Two years before being admitted to the Department of Neurosurgery, the 1st patient, female, underwent right radical mastectomy for breast cancer followed by chemotherapy treatment and subsequently developed peripheral neuropathy (as shown in the results from electroneurography (ENG) tests; Figure 3, Figure 4 and Figure 5).

Two years before vs. resection, the 2nd female patient underwent surgical treatment for melanoma and uterine cancer, received chemotherapy, and subsequently developed peripheral neuropathy. The 3rd patient, male, met the first three criteria for patient selection and undoubtedly represents the effectiveness of anastomosis inutilizing the AC versus the hypoglossal nerve. The 4th patient, female, was diagnosed 12 years before vs. resection with peripheral neuropathy secondary to Hashimoto thyroiditis and chronic hypothyroidism. The ages of the patients ranged from 61 to 65 years (mean, 63 years). All patients developed HB grade VI facial palsy after vs. resection; subsequently, they underwent AC and FN end-to-end anastomosis (between 2015 and 2018) and required more than 1.5 y (a mean of 27 months) to complete the follow-up program. The time elapsed between tumor resection and the reconstructive surgical technique ranged from 6 to 26 months (a mean of 16 months).

### 2.2. Surgical Technique (Video 1)

#### 2.2.1. Patient Positioning

**Figure 6:**
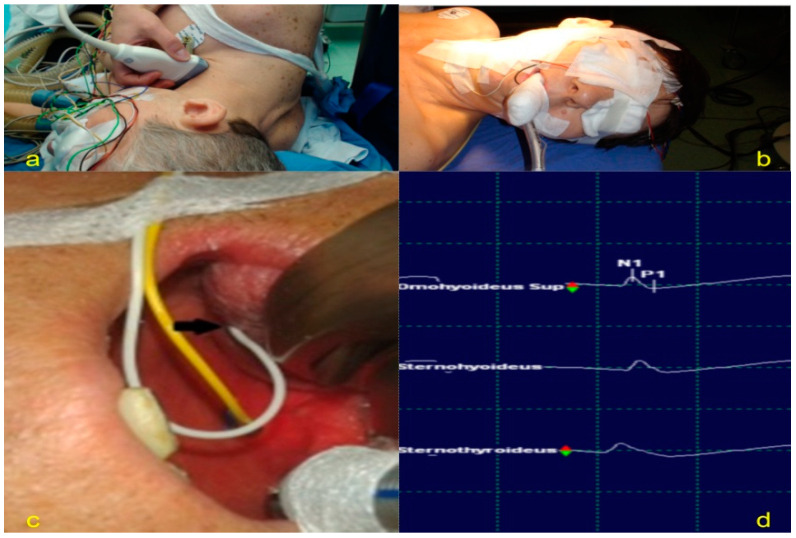
Patient positioning: (**a**) Left lateral decubitus. (**b**) Needle electrode insertion for FN monitoring (o. oris and o. oculi). (**c**) Electrode positioning for hypoglossal nerve monitoring (glossus). (**d**). Recorded potentials during mapping through electrodes positioned in the omohyoideus, sternohyoideus, and sternothyroideus muscles.

General anesthesia was performed according to the intraoperative neurophysiological monitoring protocol: Train-of-four (TOF) monitoring and total intravenous anesthesia (TIVA), focused on FN function and AC muscle innervation. The patients were positioned in the lateral decubitus position for better dissection of the anatomical structures, and an axillary roller was used to prevent injury to the brachial plexus (Figure 6a).

#### 2.2.2. Neurophysiological Monitoring

**Figure 7:**
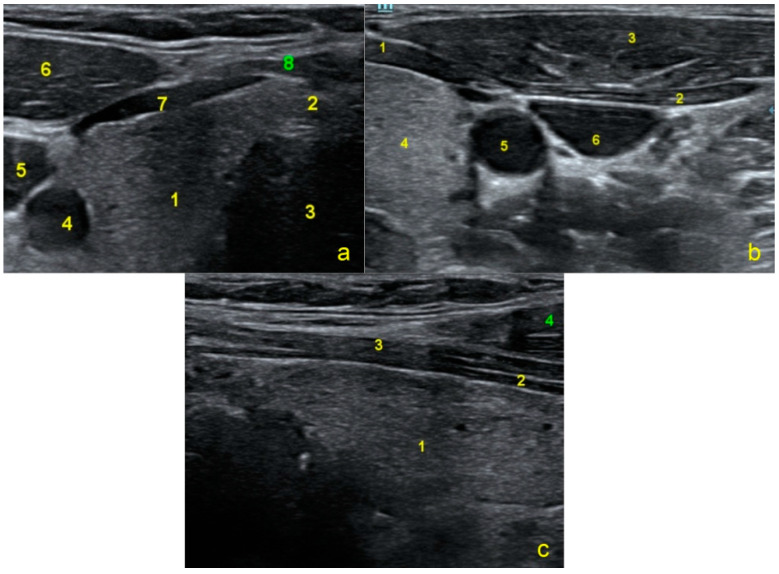
(**a**) Axial view at the level of the thyroid: Right thyroid lobe (1), isthmus (2), trachea (3), common carotid artery (4), internal jugular vein (5), sternocleidomastoid (6), sternothyroid (7), and sternohyoid (8). (**b**) Axial view showing the sternothyroid (1), omohyoid (2), sternocleidomastoid (3), thyroid (4), common carotid artery (5), and internal jugular vein (6). (**c**) Longitudinal view at the level of the thyroid shows the right thyroid lobe (1), sternothyroid (2), sternohyoid (3), and sternocleidomastoid (4).

An XLTekProtektor IOM system was used for monitoring to guide the surgeon through triggered electromyography (EMG) for mapping the nerves during dissection and through free-run EMG for avoiding lesions. Needle electrodes were placed onthe surface of the orbicularis oris and orbicularis oculi for the FN to demonstrate the lack of innervation (Figure 6b). The other electrodes were placed in the tongue for the hypoglossal nerve (Figure 6c) and in the omohyoideus superior, sternohyoideus, and sternothyroideus for the AC (Figure 6d) using ultrasound guidance (Figure 7a–c).

#### 2.2.3. Surgical Technique

**Figure 8:**
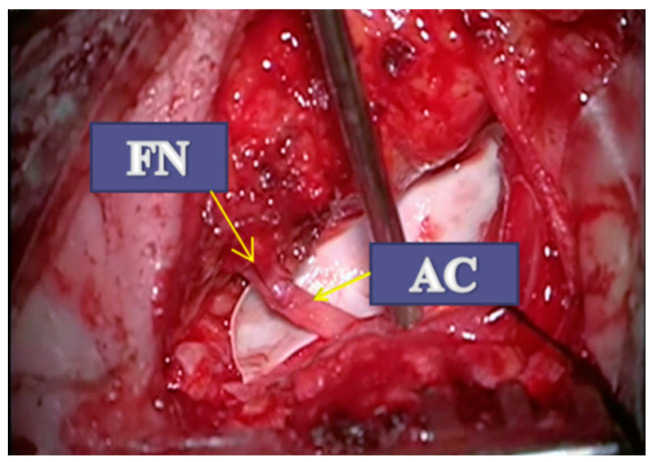
Anastomosis between the ansa cervicalis (AC) and facial nerve (FN).

A retroauricular skin incision was made on the afflicted part of the face, extending down at the lateral cervical region up to 2–3 cm below the inferior edge of the mandible. The great auricular nerve was preserved as an option for an interposed nerve graft, although this was not necessary. The styloid process represented a “marker” forFN identification. Mastoidectomy was completed utilizing a rapid drill, and the FN was identified arising from the styloid foramen. The FN was then exposed to the temporofacial division (pes anserinus) through antegrade dissection. The sternocleidomastoid muscle was typically posteriorly withdrawn, and the digastric muscle was exposed and retracted superiorly for identifying the hypoglossal nerve, which lies underneath. The AC is a component of the carotid triangle, situated between the internal jugular vein (IJV) and internal carotid artery (ICA); it was dissected as distally as possible and sectioned before dividing it into its three branches. The AC was then mobilized superiorly for epineural neurorrhaphy with FN, severed before the level of the styloid foramen, and mobilized inferiorly, thus mitigating unwanted injuries. Under an operating microscope, neurorhaphy was performed using three 9/0 nylon sutures (Figure 8). Closure started after meticulous hemostasis, and drainage was not necessary. Because facial reinnervation began some time later, tarsorrhaphy was considered a necessary surgical procedure.

### 2.3. Follow-Up Protocol

The follow-up protocol consisted of a multidisciplinary approach: Imaging examinations, clinical evaluation, and neurophysiological tests. The clinical assessment included a brain MRI after3 months,6 months, and 1 year to evaluate tumor regrowth. Patients were encouraged to undergo external electrical stimulation of the facial muscles under the guidance of a trained therapist, starting 3 months after the anastomosis. EMG was performed every 6 months during the first two years and then once per year. After the detection of initial reinnervation potential, we initiated FN conduction studies. ENG was also used to evaluate polyneuropathy. All neurophysiological tests were performed using an EMS Biomedical 4- channel EMG system. For patients with prior oncological pathology, periodic evaluations were conducted to determine the presence of a recurrent tumor or metastasis. These period evaluations included clinical examinations, brain MRI, and positron emission tomography (PET). Patients were encouraged to contact the hospital if they experienced pain, numbness, or other unusual symptoms in the cervical or facial areas. Steroid treatment was performed forall four patients after surgery, namely, 2mg/kg for five days on the intravenous route and then2mg/kg for ten days (per os route), considering additional neurological monitoring.

## 3. Results

**Figure 9:**
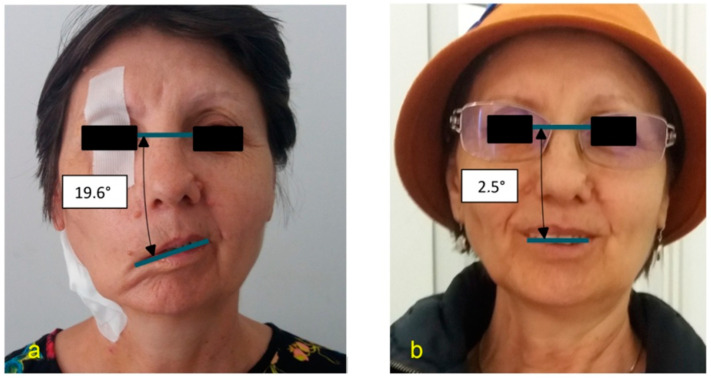
Measurements of facial asymmetry (**a**), HB grade VI right facial palsy—first patient before discharge after anastomosis, and symmetry (**b**), HB grade III right facial palsy—first patient after 16 months using the angle of the interpupillary and the intermodiolar line (pupillo-modiolar angle).

From Apr 2015 to Jan 2018, four patients (three female andonemale) were included in this descriptive study and underwent surgery for vs. through a retrosigmoid approach. Gross total tumors wereresected under an operating microscope. All four patients developed HB grade VI facial palsy after Koos grade IV vs. surgery (Table 1), based on ENoGtests. Prior to admission, the firstpatient underwent surgery for breast cancer and the second patient underwent surgery for uterine cancer and melanoma; both patients received established chemotherapy treatment and developed CIPN. The third female patient had posthypothyroidism polyneuropathy. For these patients, a diagnosis of CIPN was obtained using ENG and the clinical version of the Total Neuropathy Score (TNSc, Table 2). After anastomosis was performed, none of these patients developed postoperative complications, such as additional nerve injuries, infections, or postoperative hematoma. The first two patients reported an early initial facial reinnervation 7 months after the anastomosis. Therefore, unforeseen satisfying outcomes were observed within 7 to 9 months following the surgical procedure. The last two patients needed more than 12 months to experience clinical results. Consequently, the first two patients could spontaneously smile (HB grade III facial palsy, Figure 9a,b), and the last two patients could raise the corners of their mouths with mild synkinesis (HB grade IV facial palsy). Emotrics software (Massachusetts Eye and Ear Infirmary, Boston, MA, USA) was used to measure the smile angle, commisure excursion, marginal reflex distance, and commisure height deviation [4].

An angle of 0° represents perfect symmetry.

**Figure 10:**
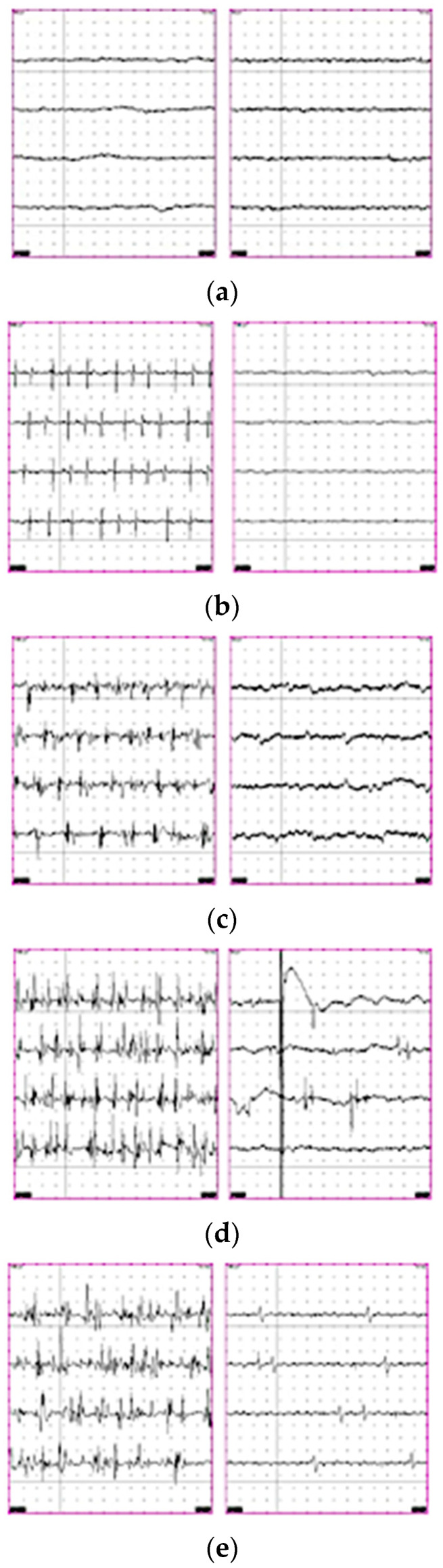
Progression of reinnervation in o. oris (left panels) and o. oculi (right panels). (**a**) Initial postoperative EMG: No activity. (**b**). O. oris with slight reinnervation. (**c**). O. oris with moderate activity. (**d**). O.oculi with slight reinnervation. (**e**). Cvasi-completely reinnervated o.oris and partially reinnervated o. oculi.

The anastomosis resulted in the gradual restoration of the facial muscles, as the initial reinnervation starts from the lower part of the face; because of the coexistence of trigeminal nerve sensory deficits, management of the eye included tarsorrhaphy, ophthalmic ointments, and protective taping. Therefore, none of the patients developed postoperative corneal opacities due to keratitis. EMG recorded fine-eye muscle movements more than 18 months after anastomosis. Although the male patient did not meet all the inclusion criteria for this study, he was included as one of the first patients treated surgically for facial paresis utilizing the aforementioned surgical technique while corresponding to a unique component. Obstructive hydrocephalus appeared immediately 8 months after the anastomosis; therefore, endoscopic ventriculocisternostomy of the thirdventricle was performed as the initial alternative. The patient quickly developed a cerebrospinal fluid (CSF) fistula at the retromastoid incision level, created for the retrosigmoid approach. We inserted lumbar drainage for 7 days; however, the CSF fistula continued. After threenegative microbiological and normal biochemical CSF samples, a contralateral ventriculoperitoneal shunt was placed, yielding a positive outcome. Tongue atrophy or masticatory disorders did not emerge in any of these patients. Figure 10a–e show the evolution of reinnervation during the periodic EMG evaluations for patient 2 (the second female patient).

**Figure 11:**
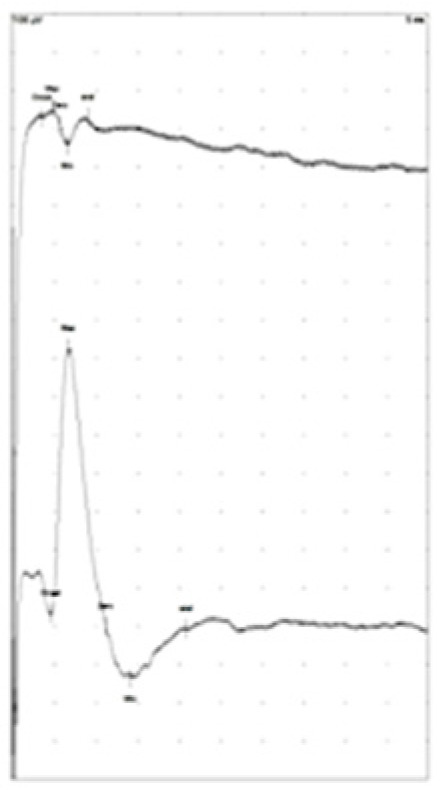
Compound motor action potential (CMAP) obtained after stimulation of the reinnervated facial nerve in the vicinity of the mandibular angle and recorded over the nasalis muscle (middle face). Notice the difference in amplitude; compared with the healthy side, the CMAP is quantifiable.

After the EMG follow-up, nerve conduction studies were performed. Figure 11 shows an ENG trace for the firstpatient.

## 4. Discussions

Although intraoperative preservation of facial nerve integrity is key to acceptable postoperative FN function [30], 30–50% of patients develop permanent FN deficits after gross total resection of vs. [31,32]. Among other predictive factors that greatly impact postoperative FN function [33], tumor size is crucial. Koos [34] grade IV is correlated with high HB [35] grade facial palsy postoperatively. In this study, all four patients had Koos grade IV vs. and presented with HB grade VI facial palsy after resection.

Depending on the long-term outcome, oncological patients are often excluded from clinical trials. According to the Guidelines Regarding the Inclusion of Cancer Survivor & HIV-Positive Individuals in study tests, patients with progression-free status should not be excluded from studies unless there is a high possibility of previous malignancy reappearance and overall survival is a study endpoint [36]. Although anastomosis was performed more than 2.5 years after tumor resections, the first two patients presented with no tumor recurrence; additionally, according to their PET/CT reports at the time of admission, no findings of vs. were seen on brain MRIs with contrast dye. Considering the diagnoses of CIPN (for the first two female patients) and peripheral neuropathy due to chronic hypothyroidism (the third female patient), the patients were informed of the possible complications and the study limits before providing written consent for anastomosis. CIPN, known for its compromised axonal transport in nerve conduction, was assessed according to electrophysiological data. Both patients presented with TNScgrade 2 (symptomatic form).

Despite the advantages and disadvantages of different surgical techniques for improving facial muscle tone [4,5,37], there are tantalizing similarities but also substantial differences between different anastomoses [38,39]. Use of the XII cranial nerve for direct neurorrhaphy with the VII cranial nerve could lead to tongue motor function impairment as an additional undesirable effect for patients already with peripheral neuropathy. Therefore, considering the axonal damage due to peripheral neuropathy, the AC was highly suitable for performing the anastomosis, compared to the hypoglossal nerve. Intraoperative or immediate postoperative local complications that could have an unfavorable impact on facial restoration include tensioned anastomosis, incidental resection of the digastric muscle (posterior belly), local hemorrhage, and wound infection. The digastric muscle can protect the anastomosis; therefore, an important step during intraoperative dissection is the preservation of this muscle.

Hydrocephalus is a systemic condition that might affect motor impulse transmission throughout the reinnervation phase. The ansa cervicalis, the superior root, is associated with the hypoglossal nerve; consequently, reinnervation is accomplished via this nerve. Increased pressure within the posterior fossa might have contributed to this particular case. The male patient developed hydrocephalus 8 months after the anastomosis. The HB grade IV facial palsy that developed in the patient 2 years after the anastomosis might additionally be related to increased intracranial pressure. Although the patient did not develop acute phase hydrocephalus, the duration between VCS and shunt insertion was approximately 2 months, during which the patient developed a CSF fistula.

Studies have shown a remarkable distinction regarding axonal counts and nerve diameters at the facial level, with the right side of the face possessing far larger values [40]. Consequently, we might credit the difference between our patients’ grades of HB facial palsy (grade III on the right side versus grade IV on the left side) to this specific anatomical observation. Despite having utilized the same dissection procedure to identify the nerves as well as the same neurorrhaphy procedure for all four patients, there was no association between the duration of facial nerve paralysis before neurorrhaphy and the postoperative outcome; the postoperative outcomes of the patient with the shortest duration (6 months) and that of the patient with the largest duration (26 months) were similar.

Kukwa et al. performed a surgical technique with outstanding immediate results for avoiding keratitis through temporal muscle transposition on the affected side. In regard to patients diagnosed with peripheral neuropathy, our team strongly recommends avoiding utilizing the XII cranial nerve as a motor resource for neurorrhaphy with the facial nerve, as it is suitable to use the AC to prevent additional nerves damage, along with temporal muscle transposition; hence, tarsorrhaphy will certainly no longer be necessary forthe future 20.

Over long-term follow-up (up to 3 years), the patients presented with good clinical recovery as assessed by serial electrophysiological parameter measurement. Patients with CIPN gradually achieved HB grade III facial palsy. Given the predominance of CIPN and oncological pathology, the results were surprising.

Future extensive studies will result in a more fundamental understanding of nerve regeneration mechanisms. This is a descriptive study based on four cases; thus, statistical significance could not be determined for the data. Nevertheless, it provides new information regarding facial palsy recovery in patients with polyneuropathy underlining CIPN. To the best of our knowledge, this is the first study to assess the efficiency of anastomosis between the AC and FN in patients with associated oncological pathology.

## 5. Conclusions

Anastomosis between the AC and FN is an effective and safe procedure for facial palsy restoration. Even patients with polyneuropathies induced by chemotherapy or hypothyroidism and long progression-free survival may benefit from this procedure. Further research would be beneficial to explore the value of statistical significance.

## Figures and Tables

**Table 1:** Patient data.

Patient	Age/Gender	Etiology	Associated Pathology	Duration of Palsy before the Anastomosis (Months)	Additional Procedures	H&B (Pre/Postop)	Postop Complications
1.	61/F	VS (Jun 2015) Koos grade IV(large tumor with brainstem displacement)	Breast cancer(treated surgically in 2013), CIPN—TNSc grade 2	19	Tarsorrhaphy	VI/III (right side) Total paralysis/obvious weakness but not totally disfiguring, complete eye closure with effort and symmetric at rest	-
2.	65/F	VS (Sep 2016) Koos grade IV	Uterine cancer, Melanoma (treated surgically in 2014), CIPN—TNSc grade 2	13	Tarsorrhaphy	VI/III (right side)	-
3.	64/M	VS (Apr 2015) Koos grade IV	-	26	Tarsorrhaphy	VI/IV (left side) Total paralysis/Disfiguring weakness, incomplete eye closure and asymmetric at rest	CSF leak Hydrocephalus (VCS, right VP shunt)
4.	63/F	VS (Jan 2018) Koos grade IV	Chronic hypothyroidism (2006) PN—TNSc grade 2	6	Tarsorrhaphy	VI/IV (left side)	-

**Table 2:** Total Neuropathy Score—clinical criteria.

TNSc					
Sensory symptoms	0	1	2	3	4
	None	Limited to fingers or toes	Extended to ankle or wrist	Extended to knee or elbow	Above knees/elbows
Motor symptoms	None	Slight difficulty	Moderate difficulty	Requires help/assistance	Disabled
Autonomic symptoms	0	1	2	3	4 or 5
Pin sensation	Normal	Reduced in fingers or toes	Reduced up to wrist/ankle	Reduced up to elbow/knee	Reduced above elbow/knee
Vibration sensibility	Normal	Reduced in fingers or toes	Reduced up to wrist/ankle	Reduced up to elbow/knee	Reduced above elbow/knee
Strength	Normal	Mild weakness	Moderate weakness	Severe weakness	Paralysis
Tendon reflexes	Normal	Ankle reflex (AR) reduced	AR absent	AR absent and others reduced	All reflexes absent

## Data Availability

The data are not publicly available in accordance with consent provided by participants on the use of confidential data.
